# Ultrasensitive and High‐Resolution Protein Spatially Decoding Framework for Tumor Extracellular Vesicles

**DOI:** 10.1002/advs.202304926

**Published:** 2023-11-20

**Authors:** Chi‐An Cheng, Kuan‐Chu Hou, Chen‐Wei Hsu, Li‐Chiao Chiang

**Affiliations:** ^1^ School of Pharmacy College of Medicine National Taiwan University Taipei 10050 Taiwan; ^2^ Department of Medicine College of Medicine National Taiwan University Taipei 10050 Taiwan

**Keywords:** disease diagnostics, exosomes, extracellular vesicles, pancreatic cancer, protein biomarkers, single molecule array

## Abstract

Proteins localized on the surface or within the lumen of tumor‐derived extracellular vesicles (EVs) play distinct roles in cancer progression. However, quantifying both populations of proteins within EVs has been hampered due to the limited sensitivity of the existing protein detection methods and inefficient EV isolation techniques. In this study, the *eSimoa* framework, an innovative approach enabling spatial decoding of EV protein biomarkers with unmatched sensitivity and specificity is presented. Using the luminal *eSimoa* pipeline, the absolute concentration of luminal RAS or KRAS^G12D^ proteins is released and measured, uncovering their prevalence in pancreatic tumor‐derived EVs. The pulldown *eSimoa* pipeline measured absolute protein concentrations from low‐abundance EV subpopulations. The *eSimoa* assays detected EVs in both PBS and plasma samples, confirming their applicability across diverse clinical sample types. Overall, the *eSimoa* framework offers a valuable tool to (1) detect EVs at concentrations as low as 10^5^ EV mL^−1^ in plasma, (2) quantify absolute EV protein concentrations as low as fm, and (3) decode the spatial distribution of EV proteins. This study highlights the potential of *eSimoa* in identifying disease‐specific EV protein biomarkers in clinical samples with minimal pre‐purification, thereby driving advancements in clinical translation.

## Graphical abstract

Proteins on the surface or within the lumen of tumor‐derived extracellular vesicles (EVs) play distinct roles in cancer progression. This study introduces an innovative framework for decoding the spatial distribution of EV protein biomarkers, offering unparalleled sensitivity and specificity. This innovation allows for precise quantification of EV protein concentrations in plasma, revolutionizing EV research and showcasing its clinical diagnostic potential.

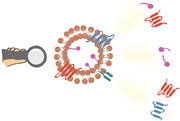

## 1 Introduction

Extracellular vesicles (EVs) are heterogeneous nanosized particles enclosed by lipid bilayers that are released by all cells, including tumor cells, in the body.^[^ ^1^ ^]^ EVs serve as carriers of nucleic acids, proteins, and lipids, facilitating intercellular communication and providing valuable insights into the parental cells’ characteristics and diseases. Analyzing and “decoding” the spatially compartmentalized information carried by EVs may reveal the identities and biological features of their parental cells, known as cells‐of‐origin. This spatial decoding approach enables the detection of abnormal gene expression patterns and other biological features associated with disorders in solid tissue sites. The comprehensive cargo of EVs offers a wealth of information that surpasses what can be obtained from analyzing individual proteins alone. Tumor cells actively release EVs early in tumorigenesis, and these circulating tumor‐derived EVs play a crucial role in disease progression. Among the conventional liquid biopsies used in oncology, including circulating tumor cells (CTCs), circulating tumor DNA (ctDNA), and EVs, EVs offer the advantage of long‐term storage for isolation and detection.^[^ ^2^ ^]^ The abundance of EVs in the blood (≈10^9^–10^12^ mL^−1^) far exceeds the number of CTCs (<10 CTCs mL^−1^)^[^ ^3^ ^]^ and the amount of ctDNA from a given gene (38% of patients harboring fewer than 2 mutant templates per mL of plasma in pancreatic cancer^[^ ^2^, ^4^ ^]^). This significant difference in concentration highlights the potential of circulating tumor‐derived EVs as “biomarker reservoirs” that offer a great depth of information compared to CTC, ctDNA, and soluble proteins. Furthermore, EVs are actively secreted by living cells, whereas ctDNA predominantly reflects the condition of dead or apoptotic cells.^[^ ^5^ ^]^ Therefore, tumor‐derived EVs hold great promise as a minimally invasive approach for cancer diagnosis, prognosis, and treatment monitoring across all disease stages, with particular relevance to early‐stage disease when timely intervention can significantly improve clinical outcomes.

The analysis of circulating tumor EVs has been a significant challenge in the field. Circulating tumor EVs represent a minute fraction of the total pool of circulating EVs, which originate from a vast constellation of different cell and tissue types and display tremendous diversity in size and protein content. While tumor‐specific biomarkers have been established for human cancers, only a subset of these markers is present in circulating tumor EVs, and their abundance is often very low, particularly in early‐stage cancer.^[^ ^6^ ^]^ Existing methods suffer from limited sensitivity, making it difficult to detect proteins in rare populations of tumor EVs, especially in early‐stage disease. These methods often rely on extensive pre‐processing steps such as ultracentrifugation, density gradient centrifugation, and size exclusion chromatography (SEC) to enrich EVs, followed by manual assays to characterize the isolated EVs. However, this workflow is time‐consuming, labor‐intensive, and lacks precision, making it challenging to implement on a large scale in clinical settings. Moreover, these methods typically analyze EVs as a bulk population and cannot dissect the inherent heterogeneity among EV subpopulations. To overcome these limitations, there is a need to develop user‐friendly and sensitive tools that can quantify specific EV‐associated protein biomarkers in disease.

Various approaches based on antibodies and aptamers^[^ ^7^ ^]^ have been explored to analyze EV‐associated protein biomarkers. Compared to aptamers, antibodies offer several advantages: they are well‐established, commercially available, widely used in clinical applications, highly specific to target antigens, and resistant to nuclease degradation. These strengths of antibodies make them ideal tools for capturing EVs in biofluids. However, antibody‐based methods face challenges posed by the presence of soluble forms of these proteins in the blood.^[^ ^8^, ^9^, ^10^ ^]^ Additionally, the non‐specific binding of reagents and intrinsic noise within complex clinical samples can introduce background signals that confound the study of EV‐associated proteins, particularly for rare target proteins. One distinguishing feature of EV‐associated proteins is their sublocalization within discrete spatial compartments. They can be exposed on the outer surface of EVs, anchored to the inner membrane leaflet, or present within the EV lumen.^[^ ^11^ ^]^ Proteins from different compartments can exhibit distinct biochemical properties and play complementary roles in cancer progression.^[^ ^12^, ^13^, ^14^ ^]^ Luminal proteins, for instance, can include mutant tumor suppressor proteins, oncoproteins, and key signal transduction mediators, making them potentially highly specific cancer biomarkers.^[^ ^15^ ^]^ Although techniques such as mass spectrometry can theoretically detect luminal proteins, their application directly to blood samples is challenging due to the exceedingly high levels of other plasma proteins (e.g., albumin, immunoglobulins) relative to EV‐associated proteins. Therefore, unique strategies are necessary to accurately characterize these proteins based on their sublocation within EVs.

**Scheme 1:**
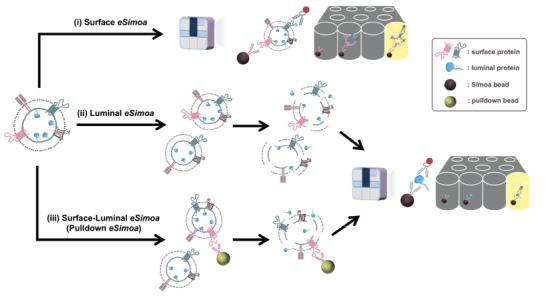
Workflow of the EV single‐molecule array (*eSimoa*) framework. The *eSimoa* framework combines EV isolation with high‐throughput Simoa technology to profile EV protein biomarkers with exceptional sensitivity and specificity. The *eSimoa* framework comprises three complementary pipelines that enable the spatial decoding of EV proteins. Pipeline (i) focuses on surface *eSimoa*, capturing and detecting two EV surface proteins. Pipeline (ii) focuses on luminal *eSimoa*, analyzing EV luminal proteins. Pipeline (iii) focuses on surface‐luminal *eSimoa* or pulldown *eSimoa*, integrating the surface and luminal *eSimoa* approaches by selectively targeting a subpopulation of EVs with a specific surface protein using pulldown beads, followed by the analysis of luminal proteins within this subpopulation.

To surmount these obstacles, we established a groundbreaking framework called EV single‐molecule array (*eSimoa*) in this study (**Scheme** **1**). Established for the first time, *eSimoa* is built on three complementary and orthogonal pipelines, enabling the spatial decoding of EV protein biomarkers with exceptional sensitivity and resolution. This framework combines EV isolation with a digital, high‐throughput Simoa technology, widely recognized and employed in basic science and clinical applications. Simoa is renowned for its unrivaled sensitivity, capable of quantifying proteins at attomolar (am; 10^−18^ m) concentrations, representing an astounding million‐fold improvement over existing methods. The use of magnetic beads in Simoa facilitates easy fluid handling and allows for automation of the workflow. The first pipeline in *eSimoa*, known as “surface *eSimoa”*, captures and detects EVs based on two surface protein biomarkers. This approach ensures that only EVs harboring both surface proteins are subjected to Simoa analysis. Orthogonal to the surface *eSimoa* pipeline, the “luminal *eSimoa”* pipeline focuses on analyzing EV luminal proteins. In addition, we developed a third pipeline, called “surface‐luminal *eSimoa*” or “pulldown *eSimoa*”, which integrates the surface *eSimoa* and luminal *eSimoa* approaches. In this pipeline, EVs with a specific surface protein are initially captured, followed by the analysis of luminal proteins within this targeted subpopulation using the luminal *eSimoa* pipeline. The *eSimoa* framework revolutionizes the analysis of EV proteins by providing a comprehensive snapshot of their spatial distribution with exceptional sensitivity. Importantly, each pipeline can be directly applied to clinical samples with minimal pre‐purification steps, making it feasible for use in clinical settings at scale.

To establish the *eSimoa* framework, we optimized multiple sets of Simoa assays targeting four pivotal protein markers: CD81 and CD63 as universal EV surface proteins, RAS as a tumor‐associated protein, and KRAS^G12D^ as a specific protein associated with pancreatic ductal adenocarcinoma (PDAC). These assays were utilized to profile tumor‐derived EVs obtained from both cancer cell cultures and human plasma samples. By harnessing the innovative *eSimoa* framework, we successfully showcased the capacity to decode both EV surface and luminal proteins while accurately quantifying the absolute concentration of luminal proteins. Together, these advancements synergize to form a powerful conceptual and technological framework, offering broad capabilities for the identification of next‐generation EV biomarkers and clinical diagnosis. If generalizable, the immediate impact of *eSimoa* would lie in identifying a robust set of novel protein biomarkers that can seamlessly integrate into minimally invasive plasma‐based clinical tests for a wide array of diseases.

## 2 Results and Discussion

### 2.1 EV Harvest and Characterization

**Figure 1:**
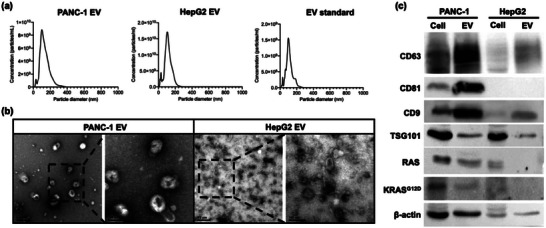
Characterization of EVs from PANC‐1 (pancreatic carcinoma), HepG2 cells (hepatocellular carcinoma), and a commercial EV standard (HBM‐HCT‐30/5, Hansabiomed). a) Nanoparticle tracking analysis (NTA) quantification and size distribution of particles contained in the sample. The modal particle size for PANC‐1 EVs was 112.1 ± 9.3 nm, with a concentration of 1.19 × 10^12^ ± 1.63 × 10^10^ particles mL^−1^. For HepG2 EVs, the modal particle size was 93.9 ± 1.9 nm, with a concentration of 1.19 × 10^12^ ± 3.49 × 10^10^ particles mL^−1^. For the EV standard, the modal particle size was 108.2 ± 7.6 nm, with a concentration of 1.07 × 10^11^ ± 2.28 × 10^9^ particles mL^−1^. b) Transmission electron microscope (TEM) imaging revealed that both PANC‐1 EVs and HepG2 EVs exhibited cup‐ or spherical‐shaped morphologies with sizes less than 200 nm. c) Western blot analysis confirmed the presence of transmembrane protein markers (CD63, CD81, and CD9), luminal protein markers (TSG101, RAS, KRAS^G12D^), and the positive control (β‐actin) in the isolated PANC‐1 EV and HepG2 EV samples, as well as their respective parental cell lysates. The amount of CD81, RAS, and KRAS^G12D^ in the HepG2 EV sample and its cell lysate was notably lower compared to the PANC‐1 samples.

To develop *eSimoa* assays, EVs from two types of tumor cells, PANC‐1 (pancreatic carcinoma) and HepG2 cells (hepatocellular carcinoma), were used. Tumor‐derived EVs were harvested and purified by ultracentrifugation from serum‐free cell culture media. These isolated EVs were characterized by nanoparticle tracking analysis (NTA), transmission electron microscope (TEM), and western blot in accordance with the MISEV guidelines.^[^ ^16^ ^]^ The size distribution analysis of EVs using NTA (**Figure** **1**a) revealed that they fell within the size range of 50 to 200 nm. The modal particle size for PANC‐1 EVs was 112.1 ± 9.3 nm, with a concentration of 1.19 × 10^12^ ± 1.63 × 10^10^ particles mL^−1^. For HepG2 EVs, the modal particle size was 93.9 ± 1.9 nm, with a concentration of 1.19 × 10^12^ ± 3.49 × 10^10^ particles mL^−1^. For the EV standard, the modal particle size was 108.2 ± 7.6 nm, with a concentration of 1.07 × 10^11^ ± 2.28 × 10^9^ particles mL^−1^. Figure 1 **b** shows TEM images of the EVs after negative staining using uranyl acetate. These EVs exhibited cup‐ or spherical‐shaped morphologies with sizes less than 200 nm. Western blot analysis (Figure 1 **c**) confirmed the presence of transmembrane protein markers (CD63, CD81, and CD9), luminal protein markers (TSG101, RAS, KRAS^G12D^), and the positive control (β‐actin) in the isolated EV samples, as well as their respective parental cell lysates. Interestingly, western blot analysis revealed that CD81 was undetectable in HepG2 cells and EVs, which is consistent with the previous report.^[^ ^17^ ^]^ In addition, RAS and KRAS^G12D^ were also observed to be present at extremely low levels in HepG2 EVs, rendering them nearly undetectable.

### 2.2 Surface *eSimoa* Pipeline for Profiling EV Surface Protein

CD81 and CD63 have been widely recognized as prominent and characteristic surface markers of EVs.^[^ ^18^, ^19^ ^]^ To specifically quantify EVs without the interference of soluble proteins, CD81 was selected as the target protein for EV capture, while CD63 served as the target protein for EV detection. Only entities containing CD81 and CD63 proteins would be captured, detected, and quantified. To develop the EV surface assay, pairs of CD81 and CD63 reagents were prepared, including CD81 and CD63 capture beads, as well as biotinylated CD81 and CD63 detector antibodies. These reagents were examined by generating calibration curves using CD81 and CD63 recombinant proteins (Figure S1a,b, Supporting Information), using CD81 and CD63 recombinant proteins. The limit of detection (LOD) values for the CD81 and CD63 Simoa assays were calculated to be 2.39 pg mL^−1^ and 0.91 pg mL^−1^, respectively.

**Figure 2:**
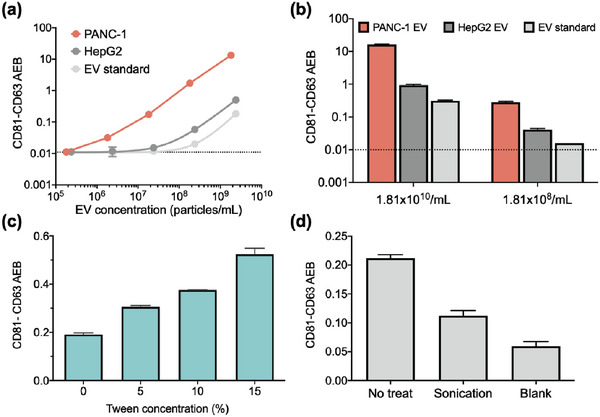
Surface *eSimoa* pipeline. a) Standard curves of the CD81‐CD63 *eSimoa* assay using PANC‐1 EVs, HepG2 EVs, and the EV standard are shown. The LOD values of the assay were calculated to be 1.2 × 10**^5^** EV mL^−1^, 3.5 × 10^6^ EV mL^−1^, and 3.1 × 10^7^ EV mL^−1^ for PANC‐1, HepG2 EVs, and the EV standard, respectively. b) The CD81‐CD63 *eSimoa* assay was used to measure PANC‐1 EVs, HepG2 EVs, and the EV standard at two different EV concentrations. The results consistently showed that PANC‐1 EVs exhibited the highest CD81‐CD63 signals compared to HepG2 EVs and the EV standard. c) The EV standard was treated with various concentrations of Tween‐20 surfactant and the CD81‐CD63 signal was measured. d) The EV standard was ultrasonically treated and the CD81‐CD63 signal was measured. All measurements were performed in duplicate. The dotted lines locate the LOD of the assay.

Once the reagents were confirmed, the CD81‐CD63 *eSimoa* assay was developed and validated using EVs derived from two types of tumor cells (PANC‐1 and HepG2) and a commercial EV standard (HBM‐HCT‐30/5, Hansabiomed) purified from the plasma of healthy individuals. Briefly, the EVs were incubated with CD81 capture beads, followed by the addition of biotinylated CD63 detector antibodies. EVs captured on the beads were then detected using an anti‐CD63 detector. The resulting complexes formed by the bead‐EV‐detector were incubated with SBG, and the mixture was loaded onto the microwell array. Within the microwell array, the catalytic reaction between SBG and RGP occurred in a confined manner. The instrument (HD‐X) is able to detect a progressively increasing fluorescent signal when a complex containing magnetic bead‐EV‐detector‐SBG is loaded into the well. The Simoa signal represents the level of CD81^+^CD63^+^ EVs. To confirm if the EV levels could be accurately represented by the CD81‐CD63 signal, PANC‐1 EVs, HepG2 EVs, and the EV standard were serially diluted to appropriate concentrations to generate calibration curves. The results demonstrated that the CD81‐CD63 signal increased with increasing EV concentrations, confirming that the CD81‐CD63 signal could be used as an indicator of EV concentration. The LOD values of the assay were calculated to be 1.2 × 10^5^ EV mL^−1^, 3.5 × 10^6^ EV mL^−1^, and 3.1 × 10^7^ EV mL^−1^ for PANC‐1, HepG2 EVs, and the EV standard, respectively (**Figure** **2**a). These findings suggested that PANC‐1 EVs had a higher subpopulation of CD81^+^ CD63^+^ EVs compared to HepG2 EVs and the EV standard.

To validate our findings, we measured the CD81‐CD63 signal in PANC‐1 EVs, HepG2 EVs, and the EV standard at two different EV concentrations. The results consistently demonstrated that PANC‐1 EVs exhibited remarkably higher CD81‐CD63 signals compared to HepG2 EVs and the EV standard (Figure 2b). It is worth noting that under normal conditions, HepG2 cells might secrete apolipoprotein B (ApoB).^[^ ^20^, ^21^ ^]^ However, NTA is unable to distinguish between EVs and other particles within the size range of EVs, including lipoproteins.^[^ ^22^, ^23^ ^]^ Therefore, the EV concentration determined by NTA for HepG2 EV might partially include contamination from lipoproteins co‐isolated with the EVs during the ultracentrifugation process. The absence of CD81 and CD63 in lipoproteins may provide another explanation for the lower CD81‐CD63 signal observed in HepG2 EVs compared to PANC‐1 EVs. This explanation is further supported by the TEM images which showed an increased presence of non‐EV particles with sizes similar to EVs in the HepG2 EV sample compared to the PANC‐1 EV sample (Figure 1b). Additionally, NTA has limitations in terms of accuracy and reproducibility. For instance, the concentration measurement by NTA is compromised by size detection limits, which can vary depending on the devices used.^[^ ^24^ ^]^ Compared to NTA, the CD81‐CD63 *eSimoa* assay offers a robust measurement of EV concentration through immuno‐recognition of both CD81 and CD63, which is not susceptible to interference from lipoproteins.

The CD81‐CD63 signal depends largely on the morphology and structure stability of EVs, as both proteins must colocalize on intact EVs or EV fragments to generate signals. A previous study^[^ ^25^ ^]^ demonstrated that EVs from another pancreatic cancer cell line (MiaPaCa) exhibited resistance to lysis effects at low concentrations of Tween‐20 surfactant (1%, 2%, and 5%, referred to as Tween). However, treatment with 10% Tween resulted in the lysis of apoptotic bodies and microvesicles, followed by exosomes, which were lysed at 15% Tween. Based on that study, we hypothesized that by leveraging the use of surfactants to treat EVs, the sensitivity of the CD81‐CD63 *eSimoa* assay could be enhanced. Our reasoning was that the surfactant would induce the rupture of the lipid bilayer of EVs, leading to multiple EV fragments remaining with both CD81 and CD63 and thus increasing the binding events between antibodies and proteins. To test this concept, we treated the EV standard with various concentrations of Tween and measured the CD81‐CD63 signal. A 2.7‐fold increase in the signal was observed at 15% Tween compared to the signal without Tween (Figure 2c). Although the signal increased gradually with the rising surfactant concentration, excessively high surfactant concentrations might not guarantee further signal enhancement. This is because at extreme fragmentation levels, the probability of CD81 and CD63 being colocalized on the same fragment and subsequently captured and detected could decrease. To examine this assumption, we subjected the EV standard to ultrasonic treatment and analyzed it using the CD81‐CD63 *eSimoa* assay. The CD81‐CD63 signal was significantly decreased to levels close to the background (AEB < 0.06) after 20 min of ultrasonic treatment, indicating a substantial disruption in the co‐localization of CD81 and CD63 (Figure 2d). This finding also confirms that the CD81‐CD63 *eSimoa* assay did not falsely detect soluble CD81 or CD63 proteins. Collectively, the EV fragmentation strategies could be leveraged to enhance the sensitivity of the CD81‐CD63 *eSimoa* assay only under mild conditions, such as surfactants at optimal concentrations or a quick sonication.

### 2.3 Luminal *eSimoa* Pipeline for Profiling EV Luminal Protein

#### 2.3.1 Pan‐RAS *eSimoa* Assay

**Figure 3:**
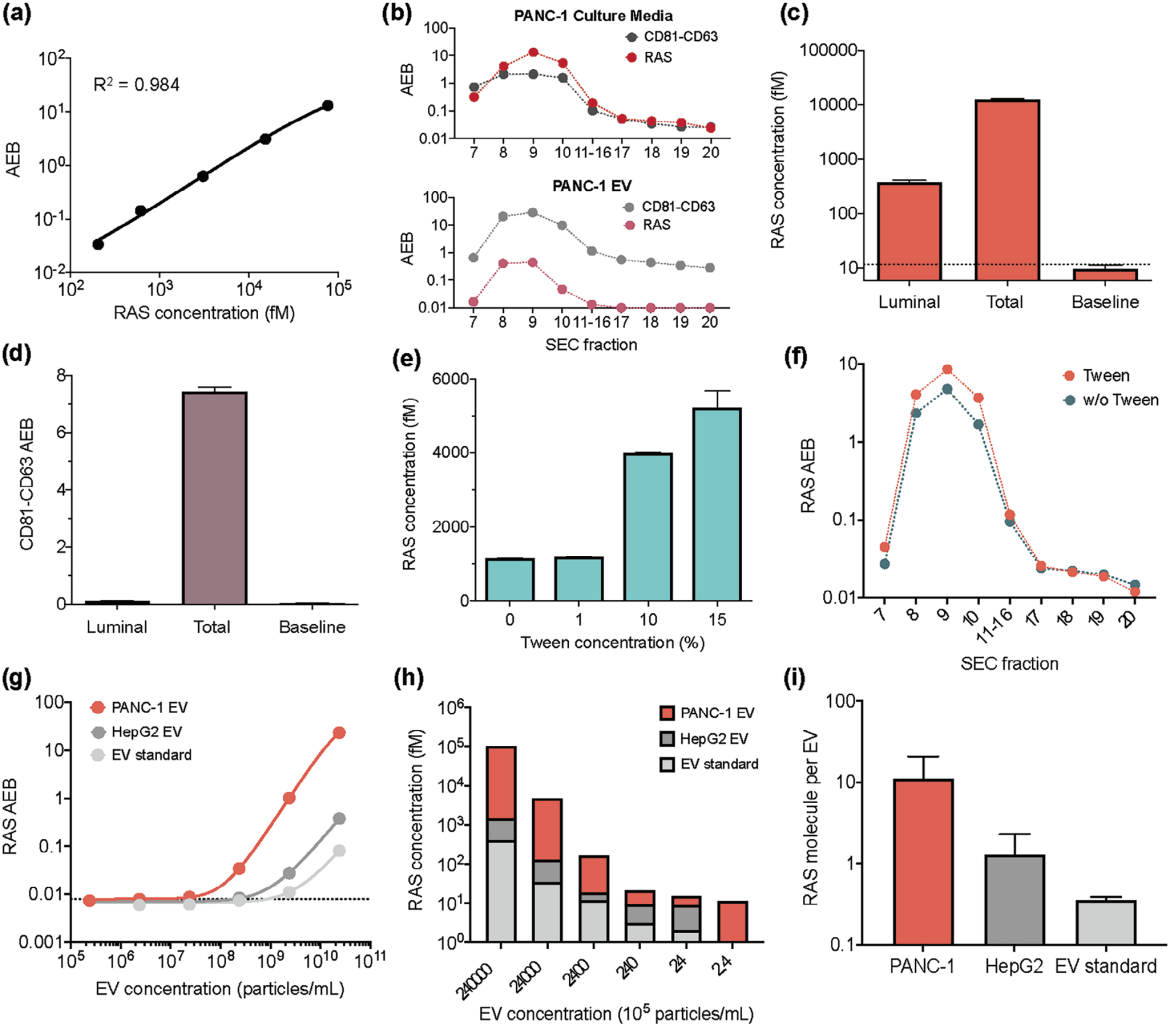
Pan‐RAS *eSimoa* assay in the luminal *eSimoa* pipeline. a) The standard curve of the pan‐RAS Simoa assay. The LOD value of the assay was 0.10 pg mL^−1^ (4.7 fm). b) *eSimoa* quantification of CD81‐CD63 and RAS levels in SEC fractions of PANC‐1 culture media (top) and the purified PANC‐1 EV sample (bottom). The pinky region (fractions 7–10) was defined as “EV fractions”. Fractions 17–20 were defined as “soluble protein fractions”. c,d) The proteinase method decodes sublocalization of RAS or CD81‐CD63 proteins within PANC‐1 EVs using the pan‐RAS or CD81‐CD63 *eSimoa* assays, respectively. Luminal: EVs were treated with proteinase K, followed by lysis of the EVs using Tween after proteinase inactivation. Total: the positive control group consists of EVs without proteinase treatment. Baseline: the negative control group in which EVs were treated with proteinase without instant proteinase inactivation. e) PANC‐1 EV samples were treated with 1%, 10%, or 15% Tween. The RAS concentrations were quantified using *eSimoa*. f) Comparison of RAS levels in the SEC fractions between two groups: PANC‐1 EVs treated with 5% Tween and without Tween treatment. g,h) RAS protein levels quantified in different concentrations of lysed PANC‐1 EVs, HepG2 EVs, and the EV standard. The LOD values were 1.7 × 10^7^ EV mL^−1^ for PANC‐1 EVs, 2.6 × 10^8^ EV mL^−1^ for HepG2 EVs, and 1.1 × 10^9^ EV mL^−1^ for the EV standard. (i) The average RAS molecules per EV were estimated to be 11.2, 1.30, and 0.36 RAS molecules per EV for PANC‐1 EVs, HepG2 EVs, and the EV standard, respectively. All measurements were performed in duplicate. The dotted lines locate the LOD of the assay.

To develop *eSimoa* assays for EV luminal (i.e., intravesicular) protein markers, we leveraged the unique features of the RAS GTPase protein that attaches to the inner leaflet of the cell membrane.^[^ ^26^ ^]^ Although the presence of RAS in the bloodstream has not been extensively studied,^[^ ^27^ ^]^ experiments in tissue culture models have suggested its insertion into the inner membrane of EVs.^[^ ^28^ ^]^ Accordingly, we hypothesized that RAS protein could be a representative marker for EVs due to its membrane localization and widespread expression in all cell types.^[^ ^29^ ^]^ Although RAS is a critical regulator of various cellular processes,^[^ ^30^ ^]^ it has not been explored as a circulating disease biomarker, and the few existing RAS immunoassays suffer from limited sensitivity, with detection limits ranging from 100–1000 pm. For instance, the suboptimal sensitivity of western blotting prevented the detection of RAS in HepG2 EVs, despite the expression of RAS in their parental cells (Figure 1c). In our previous study, we developed a pan‐RAS Simoa assay that achieved a sensitivity of 0.12 pm for quantifying pan‐RAS proteins (including KRAS, NRAS, and HRAS, as well as their mutated forms) in cells.^[^ ^27^ ^]^ To enable the detection of lower levels of RAS proteins in EVs, we further optimized the assay. Briefly, we optimized the concentration of anti‐RAS detector antibodies (0.075 µg mL^−1^) and SBG (60 pm). The optimized pan‐RAS assay presented a 25.5‐fold improvement in sensitivity (LOD: 0.10 pg mL^−1^, 4.7 fm) (**Figure** **3**a), referred to as pan‐RAS *eSimoa* assay in this study.

After confirming the presence of RAS in both PANC‐1 cells and EVs, as well as HepG2 cells (Figure 1c), we aimed to investigate the sublocalization of RAS within EVs, which is still poorly understood. To achieve this, we utilized size exclusion chromatography (SEC) to fractionate two types of samples: the culture media obtained from PANC‐1 and HepG2 cells prior to ultracentrifugation, and the purified EV samples obtained from these cells following ultracentrifugation. The fractions obtained were then analyzed using the *eSimoa* assays to profile the distribution of RAS and CD81‐CD63 proteins. For both cell types, the culture media exhibited an identical CD81‐CD63 profile to that of the purified EVs, with the majority of CD81‐CD63 proteins appearing in the early SEC fractions (fractions 7–10, referred to as “EV fractions”). This indicated that the CD81‐CD63 *eSimoa* assay specifically identified EVs and not soluble proteins present in the later fractions (fractions 17–20, referred to as “soluble protein fractions”) (Figure 3b, Figures S2 and S3, Supporting Information). The CD81‐CD63 signals increased with the concentrations of EV used for SEC (Figure S2a, Supporting Information). The percentages of EVs in each fraction were determined based on the CD81‐CD63 signal (Figures S2b and S3b, Supporting Information). The EV isolation efficiency, defined as the percentages of EVs in the EV fractions relative to the total EVs eluted in all fractions, was calculated to be 85% for PANC‐1 cells and 99.5% for HepG2 cells, indicating that the majority of eluted EVs were collected in the EV fractions. This result confirmed the successful isolation of EVs from soluble proteins in the culture media using the SEC method. Importantly, a significant portion of RAS proteins was found in the EV fractions (Figure 3b; Figure S3a, Supporting Information), indicating the association of RAS proteins with EVs. The patterns of RAS and CD81‐CD63 in the EV fractions demonstrated their overlapping distribution. In the HepG2 culture media, a bimodal distribution of RAS was observed, whereas this was less apparent in the PANC‐1 culture media (Figure 3b; Figure S3a, Supporting Information), suggesting the presence of soluble RAS proteins in the culture media. Following ultracentrifugation, the levels of RAS protein were substantially reduced to baseline levels (Figure 3b; Figure S3a, Supporting Information), indicating that ultracentrifugation effectively removed nearly all of the soluble RAS proteins.

To determine the sublocalization of EV‐associated RAS proteins within EVs, we conducted an experiment using PANC‐1 EVs. Firstly, we treated the EVs with proteinase K to degrade any proteins external to the EVs, followed by lysis of the EVs using Tween surfactant after proteinase inactivation. We then performed Simoa to measure the remaining RAS protein (referred to as luminal RAS). A positive control group, consisting of EVs without proteinase treatment, was used to measure both external and luminal RAS (defined as total RAS). A negative control group was defined as the baseline in which EVs were treated with proteinase without instant proteinase inactivation. Compared to the baseline control, we observed a significant number of luminal RAS proteins that remained even after proteinase treatment (Figure 3c). This finding suggests that these RAS proteins are located inside the EVs and are shielded from proteinase degradation by the EV lipid membrane. Previous literature suggests that RAS protein attaches to the inner leaflet of the cell membrane,^[^ ^31^ ^]^ supporting our result. To confirm these findings, we repeated the proteinase assay on PANC‐1 EVs after additional SEC purification, and the result showed a similar trend (Figure S4, Supporting Information). Initially, we hypothesized that the additional RAS proteins detected in the total RAS group, compared to the luminal RAS group, could be attributed to soluble RAS proteins that could be removed by SEC purification. However, even after SEC, these extra RAS proteins remained in the total RAS group. This led us to propose an alternative hypothesis that EV‐associated RAS proteins could be attached to either the inner or outer leaflet of the EV membrane, or exist in a soluble form encapsulated within the EV membrane. Among the EV‐associated RAS proteins co‐isolated with EVs by SEC, those attached to the external‐facing EV membrane would be susceptible to proteinase degradation, resulting in lower RAS levels compared to the group without proteinase treatment. To test this hypothesis, we performed the proteinase assay again on PANC‐1 EVs but measured the CD81‐CD63 signal instead. We reasoned that tetraspanins, such as CD81 and CD63, possess both intracellular and extracellular domains and are not present in a soluble form. Thus, they could mimic EV‐associated RAS proteins attached to the inner or outer leaflets of the EV membrane. Surprisingly, the CD81‐CD63 signal was greatly reduced to the baseline level by the proteinase, regardless of whether the EVs were lysed or not (Figure 3d). This confirmed the ability of the proteinase to degrade the extracellular domain of membrane proteins. Collectively, these findings indicate that RAS proteins could exist as EV‐associated forms (attached to the outer or inner membrane or present inside the lumen) and as soluble forms.

Next, we devised a biochemical method to release RAS proteins from EVs, based on the fact that the EV‐associated RAS proteins could be attached to the EV membrane through a covalent lipid anchor,^[^ ^31^ ^]^ or present within the EV lumen. To test this concept, we introduced various concentrations of Tween surfactant into PANC‐1 EV samples and measured RAS levels using Simoa. As the percentage of Tween increased, the amount of RAS also increased (Figure 3e). Adding 15% Tween to the EVs resulted in a 4.5‐fold increase in RAS levels. Note that RAS calibration curves were not affected in different Tween surfactant environments (data not shown), demonstrating that the change in RAS levels with Tween was not related to variations in assay sensitivity. In addition, we measured RAS levels in the SEC fractions of PANC‐1 EVs that were treated with 5% Tween. Interestingly, we observed that the Tween treatment specifically increased the RAS signal in the EV fractions, while the RAS signal remained unaffected in the soluble protein fractions (Figure 3f). This suggests that Tween treatment increased the accessibility of the EV‐associated RAS proteins to the antibodies used in the pan‐RAS *eSimoa* assay and thus increases their detection.

Although RAS is known to be highly expressed in human cells, with an average of over 2 × 10^5^ protein copies per cell,^[^ ^27^, ^32^ ^]^ the abundance of RAS proteins in EVs from different cell types has not been well characterized. To determine the RAS levels in EVs derived from different cell sources, we lysed EVs from PANC‐1 and HepG2 (tumor cells) as well as the EV standard (healthy cells). The lysed samples were serially diluted to create calibration curves for the pan‐RAS *eSimoa* assay. The LOD values were calculated to be 1.7 × 10^7^ EV mL^−1^ for PANC‐1 EVs, 2.6 × 10^8^ EV mL^−1^ for HepG2 EVs, and 1.1 × 10^9^ EV mL^−1^ for the EV standard (Figure 3g). These values not only represent the sensitivity of the assay but also reflect the RAS levels in these EVs. RAS was found to be significantly more abundant in PANC‐1 EVs compared to HepG2 EVs and the EV standard (Figure 3h). Based on the calibration curves, we estimated that, on average, there were 11.2, 1.30, and 0.36 RAS molecules per EV for PANC‐1 EVs, HepG2 EVs, and the EV standard, respectively (Figure 3i). These results are consistent with previous studies indicating that EVs derived from cancer cells contain higher levels of RAS superfamily GTPase members.^[^ ^33^, ^34^ ^]^ Moreover, RAS has been found to be highly expressed in pancreatic cancer cells compared to normal tissues.^[^ ^35^ ^]^ Contrary to our initial hypothesis that RAS could serve as a generic EV marker, our findings suggest that RAS is more prevalent in tumor‐derived EVs, indicating its potential as a tumor marker instead. This highlights the importance of considering the cell type and context when evaluating EV‐associated protein markers, as they may exhibit differential abundance and potential clinical significance.

#### 2.3.2 KRAS^G12D^ *eSimoa* Assay

**Figure 4:**
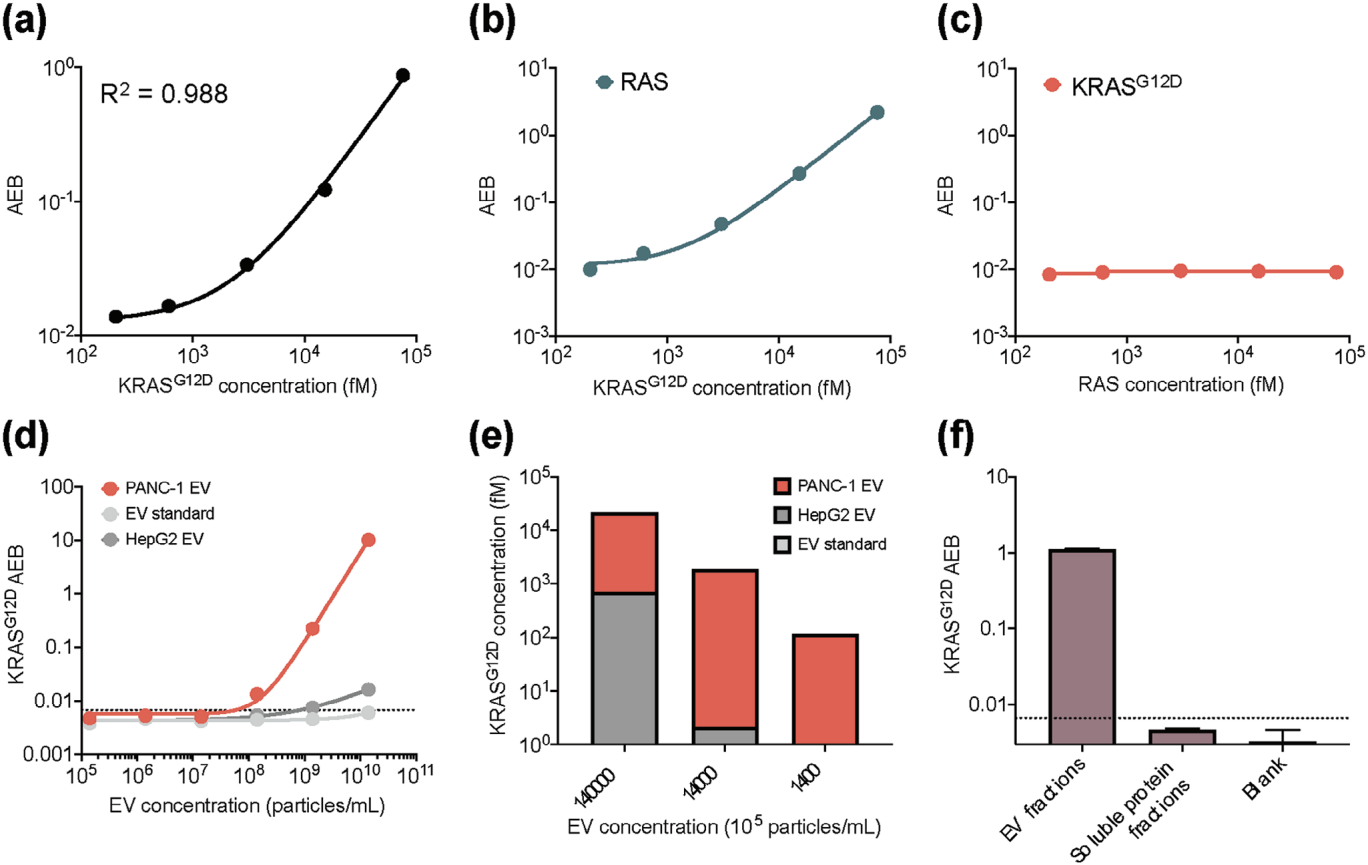
KRAS^G12D^ *eSimoa* assay in the luminal *eSimoa* pipeline. a) The standard curve of the KRAS^G12D^ Simoa assay. The LOD value of the assay was 3.18 pg mL^−1^ (151 fm). b,c) Validation of the specificity of KRAS^G12D^ Simoa assay using a cross‐testing method. KRAS^G12D^ proteins were measured by pan‐RAS *eSimoa* assay. RAS proteins were measured by KRAS^G12D^ *eSimoa* assay, which showed no detection of wildtype RAS protein even at high concentrations. d,e) KRAS^G12D^ protein levels quantified in different concentrations of lysed PANC‐1 EVs, HepG2 EVs, and the EV standard. The LOD values were 5.8 × 10^7^ EV mL^−1^ for PANC‐1 EVs and 8.1 × 10^8^ EV mL^−1^ for HepG2 EVs. No detectable KRAS^G12D^ protein was observed for the EV standard, which was as expected. f) KRAS^G12D^ signals were measured in SEC fractions of PANC‐1 EVs, which showed that KRAS^G12D^ proteins were only present in the EV fractions. All measurements were performed in duplicate. The dotted lines locate the LOD of the assay.

Mutations and hyperactivation of the RAS genes, particularly KRAS, are prevalent in approximately 20–30% of human cancer cases.^[^ ^36^ ^]^ Among the RAS proteins (including KRAS, NRAS, and HRAS), KRAS is the most commonly mutated isoform in human cancer, and its mutations have been frequently associated with fatal malignancies such as pancreatic cancer, colorectal cancer, and non‐small cell lung cancer.^[^ ^37^ ^]^ Approximately 90% of PDAC patients carry KRAS mutations,^[^ ^31^ ^]^ and the KRAS protein has been detected in EVs derived from the plasma of pancreatic cancer patients.^[^ ^38^ ^]^ Among the various KRAS mutants, the G12D mutation is the most prevalent in PDAC.^[^ ^31^ ^]^ Our western blotting result demonstrated the presence of KRAS^G12D^ protein in both PANC‐1 cells and their EVs (Figure 1c). However, currently, there is no reliable detection method available that can provide the absolute concentration of KRAS^G12D^ protein in EVs. This highlights the need for a more sensitive and specific detection approach that can quantify KRAS^G12D^ protein in EVs. To address this unmet need, we developed a KRAS^G12D^ *eSimoa* assay aiming to provide the absolute concentration of KRAS^G12D^ protein in EVs. This assay harbors good sensitivity (LOD: 3.18 pg mL^−1^, 151 fm) (**Figure** **4**a). To verify the specificity of the assay, a cross‐testing experiment was performed. Using the pan‐RAS *eSimoa* assay, we detected the KRAS^G12D^ protein, which shares a common sequence with wildtype RAS protein, indicating that the pan‐RAS *eSimoa* assay detected the common sequence (Figure 4b). However, the KRAS^G12D^ *eSimoa* assay specifically recognized KRAS^G12D^ protein only and did not detect wildtype RAS protein even at high concentrations, confirming the excellent specificity of the assay (Figure 4c).

Next, the abundance of KRAS^G12D^ protein in EVs was determined using the KRAS^G12D^ *eSimoa* assay. We lysed PANC‐1 EVs, HepG2 EVs, and the EV standard, and serially diluted the lysates to create calibration curves for the KRAS^G12D^ *eSimoa* assay. The LOD values of the assay were calculated to be 5.8 × 10^7^ EV mL^−1^ for PANC‐1 EVs and 8.1 × 10^8^ EV mL^−1^ for HepG2 EVs (Figure 4d). For the EV standard, there was no detectable KRAS^G12D^ protein, which was as expected. In a separate experiment, we quantified KRAS^G12D^ protein in these EVs at three concentrations and again observed that PANC‐1 EVs presented a significantly high abundance of KRAS^G12D^ protein compared to HepG2 EVs and the EV standard (Figure 4e). These findings suggest that the KRAS^G12D^ protein may be a promising EV marker for pancreatic cancer. To confirm that the KRAS^G12D^ protein detected in the previous experiments was associated with EVs and not in soluble forms, we performed SEC to fractionate PANC‐1 EVs and analyzed the distribution of KRAS^G12D^ protein. The results showed that KRAS^G12D^ proteins were only present in the EV fractions and not in the soluble protein fractions, confirming that KRAS^G12D^ proteins are indeed EV‐associated proteins, similar to RAS proteins (Figure 4f).

### 2.4 Validation of *eSimoa* Framework in Clinical Samples

**Figure 5:**
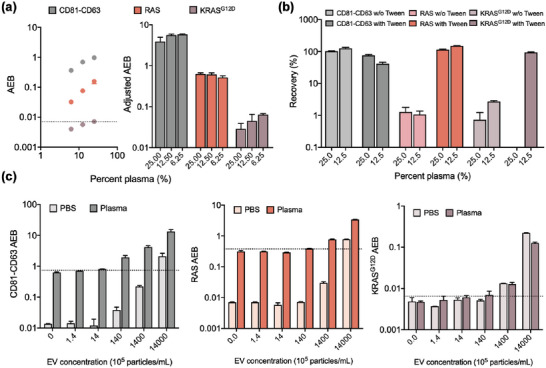
Validation of *eSimoa* framework in clinical samples. a) The signal linearity of the CD81‐CD63, pan‐RAS, and KRAS^G12D^ *eSimoa* assays was examined in serially diluted plasma samples from healthy individuals. As expected for a healthy individual, the KRAS^G12D^ protein level was below the LOD. The term “Adjusted AEB” refers to AEB values that have been corrected for the dilution factor. b) The signal recovery for each assay was evaluated in the plasma samples with the spiked PANC‐1 EVs (9 × 10^9^ EV mL^−1^). The plasma samples were diluted in PBS with or without Tween at two different dilutions. c) Plasma samples or PBS spiked with PANC‐1 EVs at varying EV concentrations were measured using the CD81‐CD63, pan‐RAS, and KRAS^G12D^ *eSimoa* assays. The lowest detectable concentrations of PANC‐1 EVs in plasma were 1.4 × 10^5^ EV mL^−1^, 1.5 × 10^7^ EV mL^−1^, and 2.3 × 10^7^ EV mL^−1^ using the CD81‐CD63, pan‐RAS, and KRAS^G12D^ *eSimoa* assays, respectively. All measurements were performed in duplicate. The dotted lines locate the LOD of the assay.

In order to validate the CD81‐CD63, pan‐RAS, and KRAS^G12D^ *eSimoa* assays in clinical samples, we attempted to measure these EV‐associated proteins in plasma samples from healthy individuals. First, the signal linearity of the assays was examined in serially diluted plasma samples. As expected for a healthy individual, the KRAS^G12D^ protein level was below the LOD (**Figure** **5**a). This result also confirmed the specificity of the KRAS^G12D^ antibodies used in the assay. The CD81‐CD63 and pan‐RAS *eSimoa* assays showed good signal linearity for all dilutions (Figure 5a), indicating that the protein levels in the diluted plasma samples fell within the dynamic range of the assays. Specifically, the pan‐RAS *eSimoa* assay demonstrated good linearity in the range of 100 to 1000 fm based on the calibration curve (Figure S5, Supporting Information).

The signal recovery rate for each assay was evaluated in the plasma samples with the spiked PANC‐1 EVs (9 × 10^9^ EV mL^−1^). The plasma samples were diluted in phosphate‐buffered saline (PBS) with or without Tween at two different dilutions. The recovery rate, expressed as a percentage, was calculated by dividing the EV protein signal measured in plasma (after baseline correction) by the EV protein signal measured in PBS and then multiplying by 100%. It provides insights into the matrix effect of the sample and the interaction of target proteins with endogenous proteins present in the samples. For the CD81‐CD63 *eSimoa* assay, the signal recovered between 70–120% of the spiked concentration under each condition, except for the sample spiked in 12.5% plasma with Tween, which showed a slightly lower recovery (Figure 5b). For the pan‐RAS and KRAS^G12D^ *eSimoa* assays, the recovery rates of the signals were poor when the EVs were spiked in the plasma diluted in PBS, but significantly increased to close to 100% with the addition of Tween (Figure 5b). This indicates that Tween facilitated the release of RAS or KRAS^G12D^ proteins from EVs, enabling their detection by their *eSimoa* assays. Moreover, Tween could also contribute to reducing the matrix effect. However, even with the addition of Tween, the recovery rate for KRAS^G12D^ protein in the 25% plasma was shown to be close to zero. This poor recovery could stem from a severe matrix effect for KRAS^G12D^ protein at this plasma dilution. Based on the results, 25% plasma will be used for the CD81‐CD63 and pan‐RAS *eSimoa* assays, and 12.5% plasma for the KRAS^G12D^ *eSimoa* assay.

Next, the sensitivity of the *eSimoa* assays was assessed in clinical samples. To simulate plasma acquired from pancreatic cancer patients at different disease stages, we spiked PANC‐1 EVs at varying concentrations in the plasma samples from healthy individuals. In the plasma sample analyzed, the *eSimoa* assays demonstrated exceptional sensitivity in detecting PANC‐1 EVs. The lowest detectable concentrations of PANC‐1 EVs were determined to be 1.4 × 10^5^ EV mL^−1^, 1.5 × 10^7^ EV mL^−1^, and 2.3 × 10^7^ EV mL^−1^ using the CD81‐CD63, pan‐RAS, and KRAS^G12D^ *eSimoa* assays, respectively (Figure 5c). These results indicate that the *eSimoa* assays have the capability to detect a small fraction, as low as five orders of magnitude lower, of tumor‐derived EVs among the estimated 10^10^ EV mL^−1^ of EVs derived from healthy tissue in the blood. Significantly, the sample matrix effect had minimal impact on the sensitivities of all three assays, as evidenced by the similar LOD values obtained in both plasma and PBS. This finding suggests that the detection of RAS and KRAS^G12D^ proteins in the assays primarily originated from the exogenously spiked EVs rather than being influenced by the components present in the sample matrix. This underscores the robust sensitivity of the *eSimoa* assays, which remained unaffected by the sample type, highlighting their suitability for accurate quantification of EV‐associated proteins in diverse clinical samples, including blood and urine.

### 2.5 Pulldown *eSimoa* Pipeline for Profiling EV Subpopulation

To profile luminal proteins in a specific EV subpopulation, we developed the third pipeline named surface‐luminal *eSimoa* or pulldown *eSimoa*. This pipeline combines the pan‐RAS or KRAS^G12D^ *eSimoa* assays with a pulldown strategy targeting a specific EV surface biomarker. To demonstrate this concept, CD81 was chosen as the target surface biomarker to pull down PANC‐1 EVs. Biotinylated antibodies specific to CD81 were utilized to label PANC‐1 EVs. Subsequently, streptavidin‐conjugated beads were used to capture the biotinylated EVs. The captured CD81‐positive EVs were then lysed and analyzed using the pan‐RAS or KRAS^G12D^ *eSimoa* assays. To optimize the pulldown efficiency, we initially determined the ideal antibody‐to‐bead ratio. Briefly, 5 × 10^8^ of PANC‐1 EVs were labeled with different amounts of biotin anti‐CD81. These labeled EVs were subsequently captured using 5 × 10^7^ of streptavidin‐coated beads. The results indicated that the optimal pulldown efficiency was achieved when using 500 ng of anti‐CD81 antibodies (Figure S6a, Supporting Information). Next, we proceeded to determine the ideal number of streptavidin‐coated beads for EV capture at this optimal antibody‐to‐bead ratio. The results showed that the optimal number of beads was 5 × 10^7^ of beads per EV sample (Figure S6b, Supporting Information). This optimized condition of antibody‐to‐bead ratio and bead number was used in the subsequent study. The pulldown efficiency, defined as the ratio of RAS measured from the captured EVs to RAS measured from the initial EVs, multiplied by 100%, was determined to be 26%. The observed fair pulldown efficiency could be attributed to the fact that not every EV is enriched with CD81.^[^ ^6^ ^]^

**Figure 6:**
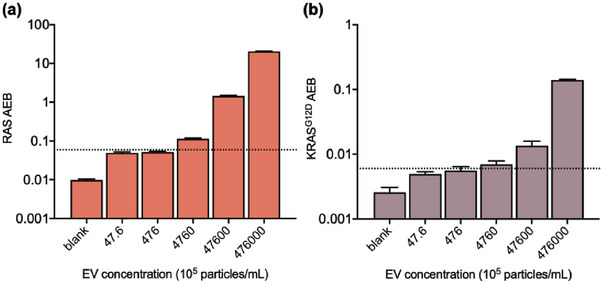
Validation of pulldown *eSimoa* pipeline in clinical samples. Plasma samples spiked with PANC‐1 EVs at varying EV concentrations were measured using the pulldown *eSimoa* pipeline. Biotinylated antibodies specific to CD81 were utilized to label EVs. Subsequently, streptavidin‐conjugated beads were used to capture the biotinylated EVs. The captured CD81‐positive EVs were then lysed and analyzed using the a) pan‐RAS and b) KRAS^G12D^ *eSimoa* assays. The lowest detectable PACN‐1 EV spiking concentration was 1.5 × 10^8^ EV mL^−1^ for RAS and 7.2 × 10^8^ EV mL^−1^ for KRAS^G12D^. All measurements were performed in duplicate. The dotted lines locate the LOD of the assay.

Finally, the pulldown *eSimoa* pipeline was validated in clinical samples. As stated above, we spiked PANC‐1 EVs at varying concentrations in the plasma samples from healthy individuals to mimic the plasma of pancreatic cancer patients. The spiked plasma samples were measured using the pulldown *eSimoa* pipeline. We observed that the levels of RAS and KRAS^G12D^ increased with the concentration of the spiked PANC‐1 EVs. Notably, the lowest detectable PACN‐1 EV spiking concentration was determined to be 1.5 × 10^8^ EV mL^−1^ for RAS and 7.2 × 10^8^ EV mL^−1^ for KRAS^G12D^ (**Figure** **6**a,b). These findings indicated that the pulldown *eSimoa* pipeline successfully detected CD81‐positive PANC‐1 EVs amidst the endogenous EV population present in the plasma.

### 2.6 Strengths and Limitations

Numerous EV analytical technologies have been previously published for the analysis of EV‐associated proteins.^[^ ^6^, ^11^, ^39^, ^40^, ^41^, ^42^, ^43^, ^44^, ^45^, ^46^, ^47^, ^48^, ^49^, ^50^ ^]^ Single‐EV analysis (sEVA) employed a fluorescence imaging technique to determine the percentage of EVs positive for specific proteins.^[^ ^6^, ^50^ ^]^ This approach required high‐resolution microscopy and the establishment of a threshold for considering vesicular fluorescence events as positive. The threshold may vary depending on the specific antibodies and fluorochromes used. Additionally, EV pre‐purification and concentration steps are required to ensure statistically acceptable EV concentrations. Most importantly, sEVA could not provide an absolute concentration of the EV markers. Other sensitive methods like Proximity Extension Assay (PEA)^[^ ^45^, ^46^, ^47^ ^]^ and Droplet‐based Extracellular Vesicle Analysis (DEVA)^[^ ^48^, ^49^ ^]^ have been proposed but have limitations. PEA lacks absolute quantification and depends heavily on proximity probes. DEVA, while sensitive, uses larger capture beads (5.4 µm) compared to Simoa beads (2.7 µm) could potentially lead to more non‐specific bindings and false positives. Another study employed PCR‐based technology where relative PCR readouts of surface proteins were reported.^[^ ^39^ ^]^ However, the analytical sensitivity of their assay was not stated. There is another study that employed a commercial ELISA kit to access EV‐associated proteins.^[^ ^11^ ^]^

#### 2.6.1 Key Advancements

Distinct from these previous studies, our work introduces a novel *eSimoa* framework that integrates two key capabilities: (1) absolute quantification of EV luminal protein concentrations with exceptional sensitivity, and (2) spatial decoding of EV proteins (surface versus luminal). The *eSimoa* framework builds upon the Simoa technology, which utilizes arrays consisting of femtoliter‐sized reaction chambers, enabling the detection of individual enzyme molecules. Simoa‐based methods have gained popularity in recent years due to their unparalleled sensitivity in detecting a wide range of protein biomarkers. Notably, the utilization of Simoa for EV protein analysis in cancer diagnostics is still in its infancy, with only five previous studies identified.^[^ ^40^, ^41^, ^42^, ^43^, ^44^ ^]^

Our work achieved three key advancements compared to these previous studies.^[^ ^40^, ^41^, ^42^, ^43^, ^44^ ^]^ First, the *eSimoa* assays exhibited superior sensitivity compared to their respective assays. Second, the *eSimoa* measured both EV surface and luminal proteins, whereas the previous studies were predominantly focused on surface proteins such as CD9, CD63, CD81, or PD‐L1. Third, our work employed the latest generation of the Simoa system (HD‐X), which differs from the systems (HD‐1 and SR‐X) used in the aforementioned studies. The state‐of‐art Simoa system used in our work is fully automated, capable of performing up to 288 measurements in approximately five hours. This system incorporates magnetic beads that facilitate fluid handling and enable workflow automation. Built on this advanced system, our *eSimoa* framework represents the most sensitive approach for quantifying EV‐associated proteins at scale. Our methodology can detect these proteins at concentrations as low as attomolar levels (am; 10^−18^ m), representing an advancement of nearly a million‐fold over existing methods. We will now discuss the strengths and limitations in more details.

##### Unmatched Sensitivity

The *eSimoa* framework demonstrated its unprecedented sensitivity to detect spiked PANC‐1 EVs in plasma at concentrations as low as approximately 10^5^ EV mL^−1^, 10^7^ EV mL^−1^, and 10^7^ EV mL^−1^, using the CD81‐CD63, pan‐RAS, and KRAS^G12D^ *eSimoa* assays, respectively. Moreover, compared with the western blot results, *eSimoa* effectively detected and quantified RAS, KRAS^G12D^, and CD81 proteins in HepG2 EV samples that were undetectable by western blotting due to suboptimal sensitivity of western blotting. The comparison between *eSimoa* and western blotting results underscores the necessity of using *eSimoa* assays to analyze non‐generic EV‐associated proteins that possibly could be false negative in western blot, particularly in the context of scarce tumor‐derived EVs. Moreover, the high resolution and broad dynamic range of the *eSimoa* assays enable simultaneous analysis of the protein from diverse EV species within the same measurement.

##### Absolute Quantification of Tumor Protein Marker in Tumor EVs

*RAS Protein*: RAS protein, one of the first discovered oncogenes,^[^ ^28^ ^]^ was chosen as the model luminal marker of EVs in this study. Our finding is consistent with the previous study showing that RAS has elevated levels in multiple cancer cell lines when compared to normal tissues.^[^ ^35^ ^]^ Recent studies have shed light on the involvement of RAS family proteins in various aspects of EV biology, including biogenesis, secretion, and cargo loading.^[^ ^28^ ^]^ Notably, proteins associated with RAS protein signal transduction have been found to be enriched in EVs derived from pancreatic cancer cell lines, relative to a human pancreatic duct epithelial cell line.^[^ ^51^ ^]^ Collectively, our findings suggest that RAS holds promise as a specific marker for tumor‐derived EVs. Moreover, the observed variations in RAS prevalence among EVs derived from different parental cells provide an intriguing opportunity for studying tumorigenesis. For instance, the increasing concentration of RAS within EVs could potentially serve as an indicator of early‐stage tumorigenesis. This capability has significant implications for early cancer detection, offering potential clinical benefits in terms of improved cancer diagnosis.

*KRAS^G12D^ Protein*: The presence of KRAS mutation in tumor tissue is a known prognostic factor for PDAC patients, regardless of whether they undergo surgical interventions.^[^ ^52^, ^53^, ^54^, ^55^, ^56^, ^57^, ^58^, ^59^ ^]^ Specifically, the G12D mutations were found to correlate with reduced survival rates in patients.^[^ ^53^, ^55^, ^59^, ^60^ ^]^ Moreover, there have been emerging targeted therapeutic strategies against specific KRAS mutant types as potential treatment options.^[^ ^37^, ^61^, ^62^, ^63^ ^]^ Therefore, knowing KRAS^G12D^ protein would hold significant clinical value not only for the diagnosis but also for guiding therapeutic decisions and monitoring treatment effectiveness.^[^ ^31^ ^]^ One study has attempted to explore KRAS mutations in endoscopic ultrasound‐guided fine‐needle aspiration (EUS‐FNA) samples and plasma ctDNA using ddPCR for early pancreatic cancer diagnosis.^[^ ^60^ ^]^ However, there are concerns associated with EUS‐FNA as it is an invasive procedure that can lead to complications and has the potential for the dissemination of tumor cells into the bloodstream during the operation. Furthermore, the sensitivity of KRAS‐positive ctDNA for pancreatic cancer detection has been limited to 20–40%.^[^ ^60^, ^64^, ^65^, ^66^, ^67^ ^]^

EVs present a compelling alternative source of KRAS mutations for two main reasons. First, EVs act as nanoreservoirs, effectively concentrating KRAS proteins and thus improving sensitivity in mutation detection. This concentration effect can be advantageous when working with low‐abundance KRAS mutations. Second, EVs are readily available in the bloodstream, allowing for their collection in a minimally invasive manner. Current studies have found a relationship between EV‐associated KRAS^G12D^ and cancers. For instance, the resection of primary localized colorectal cancer is correlated with a decrease in the levels of KRAS^G12D^ DNA copies in plasma EVs.^[^ ^68^ ^]^ KRAS oncoproteins have been frequently detected in EVs and particles derived from tumor tissue and plasma in pancreatic cancer patients.^[^ ^69^ ^]^ In the plasma of PDAC patients, 13 of the 16 EV samples were positive for either KRAS^G12D^ or KRAS^G12V 50^. KRAS^G12D^ was detectable in 45% of PANC‐1 EV according to another study.^[^ ^6^ ^]^ Despite previous efforts, no reliable method has been developed thus far to accurately measure the absolute concentration of KRAS^G12D^ protein in EVs. In our study, however, we successfully developed a minimally invasive method that enables the quantification of the absolute concentration of KRAS^G12D^ proteins in EVs with unmatched sensitivity and specificity. The concentration of KRAS^G12D^ protein holds great potential as a biomarker for evaluating the presence and progression of diseases, as well as monitoring treatment responses. It is important to note that each patient may have a unique threshold and baseline level based on their individual characteristics. By establishing personalized threshold concentrations for KRAS^G12D^ protein, clinicians can make more precise and individualized decisions in the context of precision medicine, ultimately enhancing patient care and outcomes.

##### Spatial Decoding of EV Proteins

*Surface Protein Versus Luminal Protein*: EVs play a crucial role in intercellular communication, yet their specific functions are not yet fully understood. To unravel the functions of EVs, it is essential to study their surface and luminal proteins separately, as these proteins exhibit distinct behaviors upon interaction with recipient cells. While EV luminal proteins are internalized into the cytosol of recipient cells, EV surface proteins become integrated into the plasma membrane of recipient cells.^[^ ^12^ ^]^ Changes in EV luminal protein concentrations can serve as biomarkers for treatment efficacy,^[^ ^70^, ^71^ ^]^ while EV surface proteins reflect biological responses and EV characteristics. Therefore, profiling both protein populations in EVs offers complementary insights.^[^ ^11^ ^]^ However, only a limited number of studies attempted to analyze EV luminal and surface proteins separately.^[^ ^11^, ^72^, ^73^ ^]^ This is because quantifying trace amounts of luminal or surface proteins separately requires an efficient approach for isolating EV surface and luminal protein fractions, as well as an ultrasensitive method for analysis. In this study, we addressed these requirements by employing the *eSimoa* framework, which integrates the isolation and analysis of both EV protein populations into a streamlined pipeline.

*Topology of RAS Protein*: From a clinical perspective, understanding the localization and orientation of EV surface proteins is crucial for utilizing EVs as biomarkers in various diseases. However, there is limited knowledge about the sublocalization and topology of these proteins within EV samples derived from biofluids, tissues, or cell supernatants. In this study, we employed a spatial decoding strategy that combined proteinase K treatment with SEC to uncover the sublocalization and topology of RAS proteins within EVs. Through spatial decoding, we were able to distinguish between the luminal contents of EV proteins and those present on the vesicle surface. Interestingly, in addition to identifying luminal RAS proteins, which are located on the inner membrane of EVs or present inside the EV lumen, we also observed a subset of RAS proteins that appeared to be attached to the outward membrane of EVs, suggesting their exposure to the extravesicular environment. This observation may indicate an unconventional “inside‐out” topology of lipid‐anchored RAS proteins within EVs. We hypothesize that the normal stability of the membrane was disrupted by proteinase K, leading to the reversal of the orientation of RAS proteins compared to their typical topology.^[^ ^74^ ^]^ One previous study has observed a similar “inside‐out” topology in lipid‐anchored Rab proteins, which may be attributed to alternations in the membrane lipid environment during the biogenesis of EVs, specifically during the transition from the endoplasmic reticulum to multivesicular bodies.^[^ ^75^, ^76^ ^]^ Further studies are warranted to elucidate the functional implications of this “inside‐out” topology and its relevance to EV biology and disease progression.

##### Profiling of Tumor EV Subpopulations

The pulldown *eSimoa* pipeline developed in this study sheds insight into EV diagnostics by offering a robust tool for evaluating EV‐associated RAS and KRAS^G12D^ proteins in specific EV subpopulations. This pipeline has two main applications: profiling EV subpopulations and screening potential tumor EV surface protein biomarkers. In the context of profiling EV subpopulations, CD81‐positive EVs were profiled based on their levels of RAS or KRAS^G12D^ proteins. The resulting data reflects a combination of biological and technical factors, including the number of biomarker molecules per EV, the abundance of EVs carrying the specific surface protein, and the efficiency of EV pulldown using specific antibodies. When screening tumor EV surface protein biomarkers, the magnitude of the difference in RAS or KRAS^G12D^ levels between cancer patients and control patients serves as a quantitative indicator of the potential “predictive diagnostic value” of each candidate surface protein biomarker. However, it is important to note that the enrichment of homogenous EV populations through this pipeline may result in the loss of certain EV subpopulations with diagnostic potential. This highlights a key challenge associated with sensitivity in the use of this pipeline. For instance, one previous study has shown that purification of EVs based on a single tetraspanin may lead to the loss of up to 80% of KRAS^mut^ EV.^[^ ^6^ ^]^ Specifically, the percentage of CD81‐positive PANC‐1 EVs also positive for KRAS^G12D^ is notably low. Leveraging the ultrasensitivity of *eSimoa*, we were able to successfully quantify the absolute concentrations of RAS and KRAS^G12D^ proteins even from this low percentage of EV subpopulation. However, further studies are needed to improve the detection yield, such as utilizing a pan‐tetraspanin pulldown strategy within the pulldown *eSimoa* framework.

#### 2.6.2 Clinical Significance and Prospects

Our *eSimoa* technology holds significant promise for clinical use as EV‐based diagnostics for several reasons. First, it aligns with FDA regulations, emphasizing analytical and clinical validity.^[^ ^77^ ^]^ *eSimoa*’s automation minimizes operator influence, ensuring precision. The *eSimoa* assays demonstrate reliable sensitivity and linearity in plasma samples, vital for assay validation.^[^ ^78^ ^]^ Second, for the clinical laboratory, turn‐around time and scalability of a diagnostic test are of great importance.^[^ ^79^ ^]^ *eSimoa* offers rapid high‐throughput analysis, processing 288 samples in 5 h, making it suitable for clinical laboratories. Third, while EV purity is important, practical feasibility in clinical applications takes precedence.^[^ ^80^ ^]^ Our *eSimoa* assays efficiently enrich and detect EV protein biomarkers in clinical samples with minimal pre‐purification steps. Overall, *eSimoa* meets key diagnostic requirements, including precision, sensitivity, throughput, and practical feasibility, making it highly applicable in clinical settings. Although this study primarily focused on plasma samples from healthy individuals spiked with tumor EVs and did not include clinical samples from cancer patients, future investigations utilizing the established *eSimoa* framework on EVs in clinical samples hold promise in uncovering the heterogeneity of clinical EVs and assessing the potential of EV analysis for detecting rare proteins. Based on our findings, we anticipate that *eSimoa* could be applied to the early detection of pancreatic cancer and could prove valuable in upcoming clinical trials involving patients receiving KRAS^G12D^ inhibitors or cancer vaccines targeting KRAS^G12D^. By incorporating the *eSimoa* framework into clinical studies, researchers can gain a deeper understanding of the diagnostic and therapeutic implications of EV‐associated proteins, advancing personalized medicine in the field of oncology.

## 3 Conclusions

In this study, we have introduced the *eSimoa* framework for the first time, ushering in a new era of spatial decoding for EV protein biomarkers. This innovative framework offers unmatched sensitivity, enabling the detection of EVs at concentrations as low as 10^5^ EV mL^−1^ and the quantification of absolute EV protein concentrations as low as fm. This advancement is of immense significance, considering that the proteins residing on the surface or within the lumen of EVs exhibit distinct biochemical properties and play complementary roles in cancer progression. By investigating both populations of proteins within EVs, we can gain a deeper understanding of the biological information carried by tumor‐derived EVs, which is inherited from their parental cells. Such knowledge has the potential to unlock complex mechanisms underlying cancer progression.

The *eSimoa* framework is built on three complementary and orthogonal pipelines: surface *eSimoa*, luminal eSimoa, and surface‐luminal *eSimoa* (pulldown *eSimoa*). To showcase its utility, we targeted CD81 and CD63 as EV surface proteins, while focusing on RAS and KRAS^G12D^ as EV luminal proteins of interest. We devised biochemical methods to release luminal RAS or KRAS^G12D^ proteins from EVs and used highly sensitive Simoa assays to quantify these proteins. This comprehensive approach not only provides valuable insights into the sublocalization of these proteins within EVs but also enables the identification of rare subpopulations of tumor‐derived EVs. Significantly, our findings highlight the prevalence of RAS protein in tumor‐derived EVs, suggesting its potential as a marker for tumor‐derived EVs, which could potentially be leveraged to study tumorigenesis. In parallel, KRAS^G12D^ was found to be abundantly present in EVs derived specifically from pancreatic cancer cell lines, indicating its potential as an EV marker for pancreatic cancer. Importantly, each of the three pipelines within the *eSimoa* framework can be directly applied to clinical samples with minimal pre‐purification steps, expediting EV biomarker identification, validation, and subsequent translation into a clinical blood test. The pulldown *eSimoa* pipeline, in particular, offers a powerful approach to unraveling the intrinsic heterogeneity of EVs, profiling rare subpopulations of tumor‐derived EVs, and screening for potential tumor EV surface protein biomarkers. Additionally, our study demonstrated the reliable sensitivity of *eSimoa* assays in both EVs in PBS and plasma samples, affirming their broad applicability across diverse clinical sample types.

This study showcases the application of the unmatched sensitivity and versatile technology offered by the *eSimoa* framework in the identification of promising EV protein biomarkers for pancreatic cancer. This devastating disease currently lacks reliable clinical detection methods, making the identification of such biomarkers of utmost importance. Leveraging the power of the *eSimoa* framework, we anticipate the identification of a robust panel of novel EV protein biomarkers that can be seamlessly integrated into a minimally invasive blood test for pancreatic cancer, ultimately advancing clinical translation, enabling early detection, and improving patient outcomes.

## 4 Experimental Section

### Materials and Chemicals

All antibodies and recombinant proteins used in the *eSimoa* assays are listed in Table S1 (Supporting Information). All the buffers, dye‐encoded carboxylated paramagnetic beads, and consumables used in the *eSimoa* assays were purchased from Quanterix Corporation. Dulbecco's modified Eagle's medium (DMEM), fetal bovine serum (FBS), antibiotics (10000 U mL^−1^ penicillin, 10000 µg mL^−1^ streptomycin, and 29.2 mg mL^−1^ L‐glutamine), Dulbecco's phosphate‐buffered saline (DPBS), phosphate‐buffered saline (PBS) and trypsin‐ethylenediaminetetraacetic acid (trypsin‐EDTA) (0.05%) were purchased from Gibco. Tween‐20, sodium chloride, ethylenediaminetetraacetic acid (EDTA), sodium deoxycholate, and sodium dodecyl sulfate were purchased from Sigma‐Aldrich. Bicinchoninic acid (BCA) assay protein assay kit and phenylmethylsulfonyl fluoride (PMSF) were purchased from Thermo Fisher Scientific. Tris hydrochloride (Tris HCl) was purchased from iNtRON Biotechnology. Laemmli sample buffer and Clarity Western ECL Substrate were purchased from Bio‐Rad. The antibodies used for western blotting analysis were listed in *Characterization*. Proteinase K was purchased from Invitrogen. Streptavidin‐coated silica bead was purchased from Bangs Laboratories. Sepharose CL‐6B resin was purchased from GE Healthcare/Cytiva.

### Cell Culture

PANC‐1 cells (CRL‐1469, ATCC) were purchased from American Type Culture Collection. HepG2 cells were kindly provided by Dr. Tung‐Hung Su (National Taiwan University Hospital). The cells were cultured in DMEM (10‐013‐CM, Corning, USA) supplemented with 10% fetal bovine serum and 100 U mL^−1^ penicillin‐streptomycin. The cells were grown until 80% confluence at passage numbers 10–13. Prior to harvesting EVs, the cells were cultured in a serum‐free medium for 24–48 h, and the medium was collected to harvest cell‐derived EVs. Regular mycoplasma contamination checks were performed during cell culture and prior to EV harvest.

### EV Harvest

The collected medium was subjected to centrifugation at 300 g, 4 °C for 10 min, followed by an additional centrifugation step at 2000 g, 4 °C for 20 min to remove cell debris. The resulting supernatant was carefully transferred to thin‐wall Ultra‐Clear Tubes (13.5 mL, Beckman Coulter, Inc., USA) and ultracentrifuged at 100000 g, 4 °C for 70 min using a SW41Ti rotor (Beckman Coulter, Inc., USA) to pellet the EVs. The EVs were then suspended in PBS and stored at −80 °C until further use.

### Characterization

EVs were characterized following the guidelines of the International Society for Extracellular Vesicles (ISEV).^[^ ^16^ ^]^ The size distribution and particle number were determined using nanoparticle tracking analysis (NS300, NanoSight). The NTA measurements were conducted under the following conditions: NTA version: NTA 3.4 Build 3.4.003; camera type: sCMOS; laser type: Blue 488; camera level: 14; slider shutter: 1259; slider gain: 245; FPS: 25.0; number of frames: 1498; temperature: 25 °C; viscosity: (water) 0.889‐0.889 cP; syringe pump speed: 70; detect threshold: 5. For transmission electron microscope (TEM) imaging, EV samples were absorbed on a 400‐mesh carbon‐coated copper grid at room temperature for 5 min. Thereafter, 2% paraformaldehyde was added to fix the samples, followed by negative staining with 2% uranyl acetate for 1 min. The grids were observed using a Hitachi H‐7650 TEM (Hitachi High‑Technologies, Tokyo, Japan) at an acceleration voltage of 80 kV. For western blotting, cell lysates and EV lysates were obtained by incubating samples in RIPA buffer (50 mm Tris–HCl pH 8.0, 150 mm NaCl, 5 mm EDTA, 0.5% sodium deoxycholate, 1% NP‐40, 0.1% sodium dodecyl sulfate) with a proteinase inhibitor cocktail on ice for 30 min, followed by centrifugation at 13000 rpm, 4 °C for 15 min to collect the supernatant. The total protein content was determined using the BCA protein assay kit (23225, Thermo Fisher Scientific). The cell lysates and EV lysates were heated in Laemmli sample buffer (1610747, Bio‐Rad) at 70 °C for 10 min with reducing reagent (for RAS, KRAS^G12D^, TSG101, beta‐actin) or without reducing reagent (for CD63, CD81, CD9), separated by SDS‐PAGE (10%), and transferred onto 0.2 µm PVDF membranes (1620177, Bio‐Rad). The membranes were blocked with 3% skim milk in TBS with 0.05% Tween‐20 for 1 h, followed by overnight incubation with the appropriate primary antibody at 4 °C. The target proteins were detected using HRP‐conjugated secondary antibody. After washing with TBST, the target proteins were visualized using Clarity Western ECL Substrate (1705061, Bio‐Rad) and imaged using the ChemiDoc MP Imaging System (170‐8280, Bio‐Rad). The following antibodies were used in the western blotting analysis. Primary antibodies include anti‐CD63 (1:1000, 353 039, BioLegend), anti‐CD81 (1:1000, 349 501, BioLegend), anti‐CD9 (1:1000, GTX135296, GeneTex), anti‐TSG101 (1:1000, GTX118736, GeneTex), anti‐RAS (1:1000, GTX132480, GeneTex), anti‐KRAS^G12D^ (1:1000, GTX635362, GeneTex), and anti‐beta Actin (1:10000, GTX109639, GeneTex). Secondary antibodies include goat anti‐rabbit antibody (1:5000, GTX213110‐01, GeneTex) and goat anti‐mouse antibody (1:5000, GTX213111‐01, GeneTex).

### Proteinase Assay

The purified EVs (20 µL) after ultracentrifugation were diluted to 200 µL using PBS. This solution was used for three conditions: *Condition 1. The positive control*: The EV solution (60 µL) was added with PBS (2 µL) and incubated at 37 °C for 30 min, followed by 1 h at room temperature. Next, 30% Tween‐20 (50 µL) and 100 mm PMSF (10 µL) were added to the solution. The solution was incubated on ice for 10 min before Simoa detection. *Condition 2. The negative control*: proteinase K (2 µL, a total of 40 µg) (4333793, Invitrogen) and 30% Tween‐20 (50 µL) were added to the EV solution (60 µL). The mixture was incubated at 37 °C for 30 min. Then, PMSF (10 µL) was added and incubated at room temperature for 1 h. The solution was further incubated on ice for 10 min before Simoa detection. *Condition 3. The luminal protein*: proteinase K (2 µL, a total of 40 µg) was added to the EV solution (60 µL). The solution was incubated at 37 °C for 30 min. PMSF (10 µL) was added and the solution was incubated at room temperature for 1 h. Then, the solution was added with 30% Tween‐20 (50 µL) and incubated on ice for 10 min before Simoa detection. After the final incubation, each sample was diluted in PBS to a final volume of 250 µL and measured using pan‐RAS *eSimoa* assays on the Quanterix HD‐X instrument.

### EV Isolation by Pulldown Method

First, PANC‐1 and HepG2 EVs were incubated with biotin anti‐CD81 (349514, BioLegend) at room temperature for 45 min. Then, 50 µL of streptavidin‐coated silica beads (CS01001, Bangs Laboratories) were mixed with 500 µL of PBS. The bead solution underwent centrifugation at 13000 g for 3 min. The resulting pellet was collected, resuspended, and aliquoted in PBS. The EV samples labeled with anti‐CD81 were subsequently incubated with the beads for 45 min at room temperature to isolate the EVs. The beads captured with the isolated EVs were collected by centrifuging at 13000 g for 3 min. The resulting pellet was collected and resuspended in 15% Tween‐20 to lyse the isolated EVs. After another round of centrifugation, the resulting supernatant was used for pan‐RAS *eSimoa* analysis. In the validation study in clinical samples, a healthy donor plasma sample was diluted four times for pan‐RAS assay or eight times for KRAS^G12D^ assay in PBS, followed by spiked with different amounts of EVs. The spiked plasma samples were then incubated with biotin anti‐CD81, resulting in a total volume of 190 µL. The subsequent procedure followed the same steps as mentioned earlier.

### EV Isolation by Homebrew Size Exclusion Chromatography (SEC)

The Sepharose CL‐6B resins (17016001, GE Healthcare/Cytiva) were washed three times with PBS (1:1) and stored at 4 °C before use. The columns were prepared freshly on the day of use. To prepare the column, the washed resin was poured into an Econo‐Pac Chromatography column (7321010, Bio‐Rad) to bring the bed volume (the resin without liquid) to 10 mL. Once the desired amount of resin had filled the column and the liquid had dripped through, the filter disc was immediately placed at the top of the resin without compressing it. Before adding the sample, add PBS to the column to keep the resin hydrated. The column was allowed to fully drip out once the sample was ready to add. Immediately, culture media or EV samples diluted in PBS were added to the column after the last drop of PBS. During the sample loading and fractionation, PBS was continuously added to the column (0.5 mL each time) and the fractions were collected individually for a total of 20 fractions (fractions 1–20). Finally, these fractions were diluted with PBS with or without Tween and measured using *eSimoa* assays for CD81‐CD63, pan‐RAS, and KRAS^G12D^ on the Quanterix HD‐X instrument.

### eSimoa Assays

All the Simoa capture beads and biotinylated detector antibodies were homebrewed and prepared according to the previous work.^[^ ^81^ ^]^ In the CD81‐CD63 *eSimoa* assay, EV samples were incubated with the homebrewed anti‐CD81 Simoa capture beads for 15 min. The beads were then washed with a non‐surfactant buffer to remove the unbound EVs. Subsequently, the EV‐bead mixture was incubated with the homebrewed biotinylated anti‐CD63 detector antibodies for 5 min. The resulting mixture was washed with a non‐surfactant buffer to remove the unbound antibodies. Streptavidin labeled β‐galactosidase (SBG) was then added to the mixture and incubated for 5 min, followed by several washing steps. Finally, the entire mixture was resuspended in resorufin β‐D‐galactopyranoside (RGP) before being loaded into the microwell array and analyzed on the HD‐X instrument (Quanterix). In the pan‐RAS or KRAS^G12D^ assays, EV samples were first lysed using Tween surfactant to release their luminal RAS or KRAS^G12D^ proteins. The released proteins were analyzed by their respective Simoa assays. Briefly, the released RAS or KRAS^G12D^ proteins were incubated with the homebrewed anti‐RAS Simoa capture beads or homebrewed anti‐KRAS^G12D^ Simoa capture beads for 15 min. After washing, the resulting mixture was incubated with homebrewed biotinylated anti‐RAS detector antibodies for 5 min for both assays. After washing, the mixture was incubated with SBG, washed, and mixed with RGP. Finally, the entire mixture was loaded into the microwell array and analyzed on the HD‐X instrument. All assay steps, image analyses, and calculations of average enzyme per bead (AEB) were automated. The antibodies and recombinant proteins used in the *eSimoa* assays are listed in Table S1 (Supporting Information).

### Clinical Sample Collection and Storage

Peripheral blood samples were collected from healthy donors with written informed consent according to the IRB protocols at National Taiwan University Hospital (IRB: 201312145RINB). Ethylenediaminetetraacetic acid (EDTA) vacutainer tubes (purple tube) were used for blood sample collection. The whole blood was centrifuged at 3000 rpm for 15 min. The supernatant was collected as plasma samples and stored at −80 °C until further use.

### Data Analysis

For all *eSimoa* assays, calibration curves were fit using a four‐parameter logistic (4PL) fit in GraphPad Prism 8 and used to determine unknown sample concentrations. All measurements were performed in duplicates. The limit of detection (LOD) of each assay was calculated as the concentration corresponding to three standard deviations above the background AEB. All figures were plotted in GraphPad Prism 8.

## Conflict of Interest

The authors declare no conflict of interest.

## Author Contributions

C.‐A.C., K.‐C.H., and C.‐W.H. contributed equally to this work. C.‐A.C. performed conceptualization. C.‐A.C., K.‐C.H., C.‐W.H. performed Investigation. C.‐A.C., K.‐C.H., C.‐W.H., L.‐C.C. performed data acquisition. C.‐A.C., K.‐C. H., C.‐W.H. performed data analysis. C.‐A.C. performed funding acquisition. C.‐A.C. performed supervision. C.‐A.C. performed writing of original draft. C.‐A.C., K.‐C. H., C.‐W. H. performed writing, reviewing, and editing. All authors have given approval to the final version of the manuscript.

## Supporting information

Supporting Information

## Data Availability

The data that support the findings of this study are available from the corresponding author upon reasonable request.
