# Supplementary material for: Ultrasensitive and High‐Resolution Protein Spatially Decoding Framework for Tumor Extracellular Vesicles
Source: Adv Sci (Weinh). 2023 Nov 20;11(3):2304926. doi: 10.1002/advs.202304926 (PMC10797477; doi:10.1002/advs.202304926)
Supplement: Supplementary file 1 — Supporting Information [file ADVS-11-2304926-s001.pdf]

ADVANCED SCIENCE
Open Access

Supporting Information

for *Adv. Sci.*, DOI 10.1002/advs.202304926

Ultrasensitive and High-Resolution Protein Spatially Decoding Framework for Tumor Extracellular Vesicles

Chi-An Cheng, Kuan-Chu Hou, Chen-Wei Hsu and Li-Chiao Chiang*

Supporting Information

Ultrasensitive and High-Resolution Protein Spatially Decoding Framework for Tumor Extracellular Vesicles

Chi-An Cheng,[a, ‡,*] *Kuan-Chu Hou,* [b,‡] *Chen-Wei Hsu,* [a,‡] *Li-Chiao Chiang* [a]

[a] School of Pharmacy, College of Medicine, National Taiwan University, Taipei, 10050, Taiwan

[b] Department of Medicine, College of Medicine, National Taiwan University, Taipei, 10050, Taiwan

* Corresponding author: cacheng123@ntu.edu.tw

Table of Contents

Supporting Figures……………..………………………...………………...…………...........................**S3**

Figure S1: (a) The standard curve of the CD81 Simoa assay. (b) The standard curve of the CD63 Simoa assay.

Figure S2: (a) *eSimoa* quantification of CD81-CD63 levels in SEC fractions of PANC-1 EV sample at various EV concentrations. (b) The percentages of EVs in each fraction were determined based on the CD81-CD63 signal.

Figure S3: (a) *eSimoa* quantification of CD81-CD63 and RAS levels in SEC fractions of HepG2 culture media (top) and the purified HepG2 EV sample (bottom). (b) The percentages of EVs in each fraction were determined based on the CD81-CD63 signal.

Figure S4: The proteinase method decodes sublocalization of RAS proteins within the PANC-1 EVs, which underwent additional SEC purification, using the pan-RAS *eSimoa* assay.

Figure S5: The good linearity of pan-RAS *eSimoa* assay in the range of 100 to 1000 fM.

Figure S6: The optimization of the capture efficiency for the pulldown *eSimoa* pipeline.

Supporting Table……………..………………...…………………...……….................................**S9**

Table S1. Antibodies and recombinant protein standards used in *eSimoa* assays

Supporting Figures

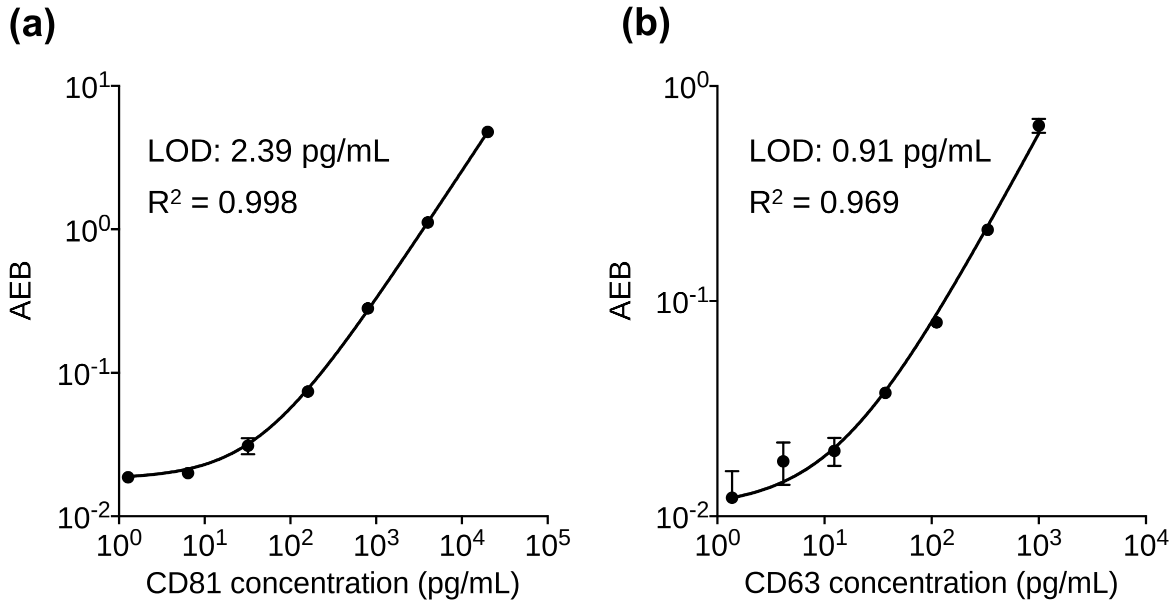

Figure S1. (a) The standard curve of the CD81 Simoa assay. The LOD value of the assay was 2.39 pg/mL. (b) The standard curve of the CD63 Simoa assay. The LOD value of the assay was 0.91 pg/mL. All measurements were performed in duplicate.

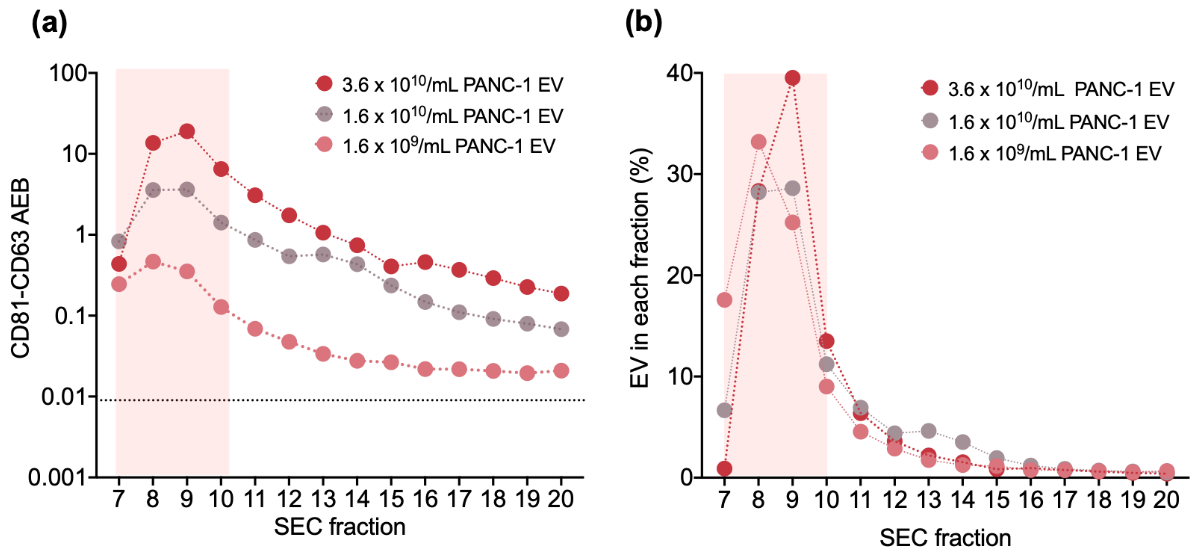

Figure S2. (a) *eSimoa* quantification of CD81-CD63 levels in SEC fractions of PANC-1 EV sample at various EV concentrations. (b) The percentages of EVs in each fraction were determined based on the CD81-CD63 signal. The pinky region (fractions 7–10) was defined as “EV fractions”. Fractions 17–20 were defined as “soluble protein fractions”. All measurements were performed in duplicate. The dotted lines locate the LOD of the assay.

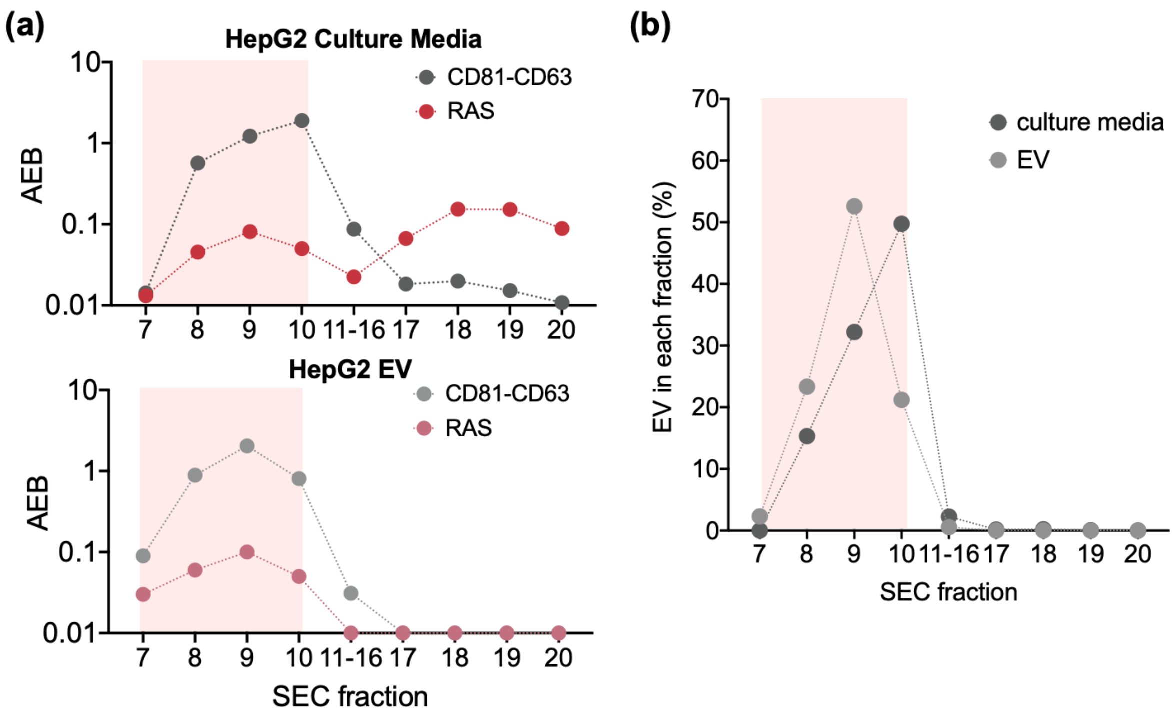

Figure S3. (a) *eSimoa* quantification of CD81-CD63 and RAS levels in SEC fractions of HepG2 culture media (top) and the purified HepG2 EV sample (bottom). (b) The percentages of EVs in each fraction were determined based on the CD81-CD63 signal. The pinky region (fractions 7–10) was defined as “EV fractions”. Fractions 17–20 were defined as “soluble protein fractions”. All measurements were performed in duplicate. The dotted lines locate the LOD of the assay.

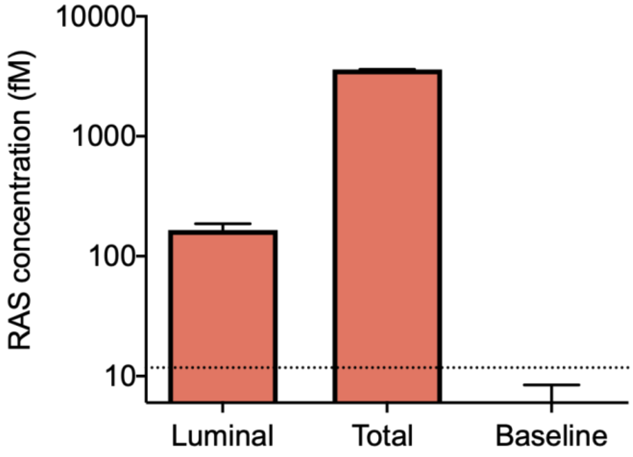

Figure S4. The proteinase method decodes sublocalization of RAS proteins within the PANC-1 EVs, which underwent additional SEC purification, using the pan-RAS *eSimoa* assay. Luminal: EVs were treated with proteinase K, followed by lysis of the EVs using Tween after proteinase inactivation. Total: the positive control group consists of EVs without proteinase treatment. Baseline: the negative control group in which EVs were treated with proteinase without instant proteinase inactivation.

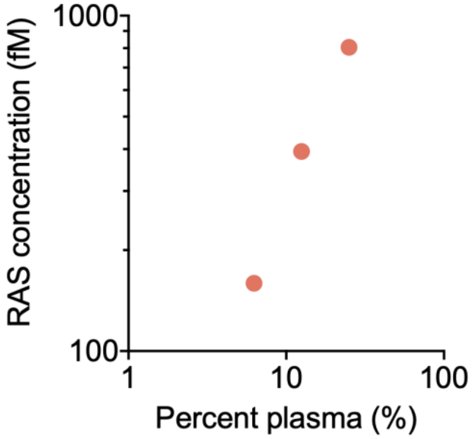

Figure S5. The good linearity of pan-RAS *eSimoa* assay in the range of 100 to 1000 fM.

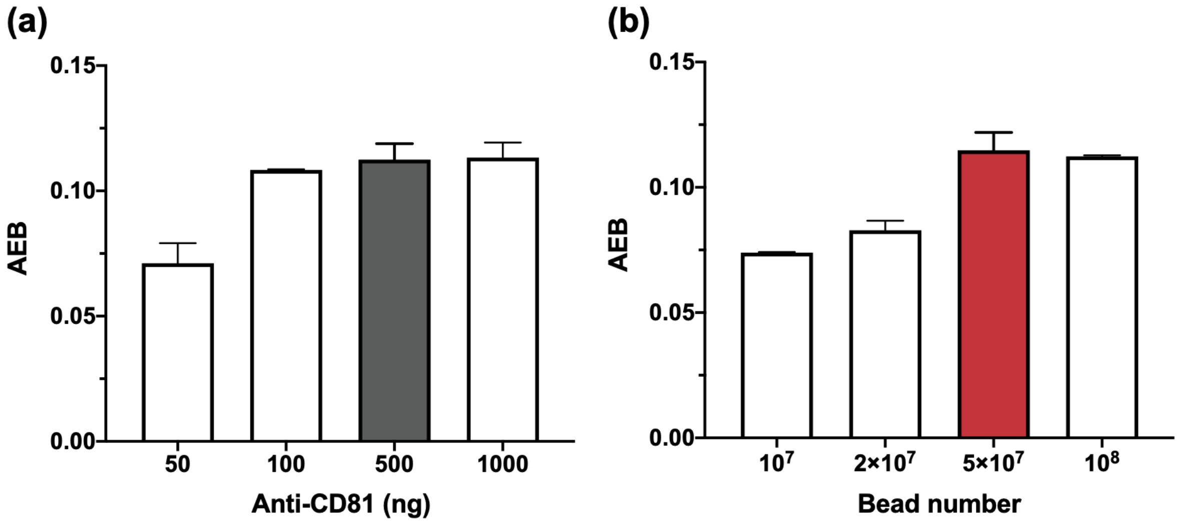

Figure S6. The optimization of the capture efficiency for the pulldown *eSimoa* pipeline.

Supporting Table

Reagent	Manufacturer
CD81 capture antibody	Abcam ab79559
CD81 detector antibody	Biolegend 349502
CD81 protein standard	Origene TP317508
CD63 capture antibody	R&D MAB5048
CD63 detector antibody	BD 556019
CD63 protein standard	Origene TP301733
RAS/ $KRAS^{G12D}$ detector antibody	Abcam ab209974
RAS capture antibody	Abcam ab55391
RAS protein standard	Abcam ab61239
$KRAS^{G12D}$ capture antibody	GeneTex GTX635362
$KRAS^{G12D}$ protein standard	Abcam ab268712

Table S1. Antibodies and recombinant protein standards used in *eSimoa* assays.
